# Acute Effects of an Ergometer-Based Dryland Alpine Skiing Specific High Intensity Interval Training

**DOI:** 10.3389/fphys.2018.01485

**Published:** 2018-10-18

**Authors:** Thomas Stöggl, Josef Kröll, Roland Helmberger, Maria Cudrigh, Erich Müller

**Affiliations:** ^1^Department of Sport and Exercise Science, University of Salzburg, Salzburg, Austria; ^2^Olympic Training Center Salzburg-Rif, Salzburg, Austria

**Keywords:** blood lactate, cardiorespiratory response, giant slalom, RPE, slalom, ski ergometer, specific testing

## Abstract

**Introduction:** To establish an alpine ski racing (ASR) specific dryland high intensity training protocol (HIT), we set out to analyze cardiorespiratory and metabolic responses of three ASR specific HIT modes using a ski ergometer compared with a running HIT.

**Methods:** Ten healthy international FIS level subjects (18 ± 1 years) performed an incremental running VO_2max_ test, three different ASR specific HIT modes [slalom (SL), giant slalom (GS), and SL/GS mix] and a running HIT with measurements of VO_2_, heart rate (HR), blood lactate, and rate of perceived exertion (RPE). The HIT protocols included 15 × 1-min intervals with >90% HR_max_ and 30 s active rest. Furthermore, one elite alpine skier performed an 8-week, 17 session HIT block using the SL/GS mixed mode.

**Results:** Running HIT resulted in greater VO_2peak_ and whole-body RPE compared with the three ASR-specific HIT modes. During all four exercise modes participants were able to reach exercise intensities high enough to be classified as HIT (>90% HR_max_ and >89% VO_2max_). Legs RPE was similar between the four HIT modes, while arms RPE was higher for the ski-specific HIT. For all studied parameters, similar results for the three skiing specific HIT modes were observed. The 8-week HIT block was feasible for the athlete and resulted in an 11% increase in VO_2max_ at unchanged peak power output.

**Conclusion:** Across all HIT protocols high cardiorespiratory and metabolic responses were achieved. Therefore, the ASR specific HIT was shown to be feasible, thus could offer new possibilities for endurance training in elite alpine skiers. It is suggested to use the SL/GS mixed mode in terms of movement variety. The reduced VO_2_ in the ski-specific modifications can be attributed to the concentric and eccentric muscle activity resulting in mechanical hindrance for O_2_ extraction. The long-term effectiveness of ASR specific HIT in elite alpine skiers needs to be proven in a future study.

## Introduction

Alpine ski racing (ASR) consists of competition runs of 45 s [e.g., slalom (SL)] up to 2.5 min (e.g., downhill) and can be categorized as high-intensity short-term endurance exercise. While technical skiing skills appear to have the greatest effect on performance, the ability to continually exhibit technical competence within a race but also through a long competitive season requires high capabilities within all physiological systems (Müller et al., 2000; Gross et al., 2009; Turnbull et al., 2009). In this context it was demonstrated that the energy contribution during ASR is based 30–65% on anaerobic and 35–70% on aerobic processes (Veicsteinas et al., 1984; Saibene et al., 1985; Andersen and Montgomery, 1988; Tesch, 1995; Grenier et al., 2013).

Maximal to near maximal heart rate (HR) values (88.9–102.5% of HR_max_; e.g., with respect to maximal HR values achieved during incremental tests to exhaustion with running or in comparison to the theoretical calculated value based on the equation 220-age) are typically attained by the end of the race in either of the four ski disciplines SL, Giant Slalom (GS), Super-G and Downhill (Karlsson et al., 1978; Veicsteinas et al., 1984; Tesch, 1995; Vogt et al., 2005; Grenier et al., 2013; Polat, 2016). However, there is discrepancy with respect to the peak oxygen uptake (VO_2peak_) reached during a run (Karlsson et al., 1978; Saibene et al., 1985; Tesch, 1995; Vogt et al., 2005; Grenier et al., 2013) with documented values between 64% in youth ASR (Grenier et al., 2013) up to 100% in the elite ASR (Tesch, 1995). The most recent study by Polat (2016) depicted 75% of VO_2max_ during GS skiing. Blood lactate concentration of 5.7 mmol L^-1^ on junior level and up to 13 mmol L^-1^ on elite level have been reported for the SL and GS disciplines (Andersen and Montgomery, 1988; Vogt et al., 2000; Grenier et al., 2013; Polat, 2016).

High intensity training (HIT) was demonstrated to be a time efficient alternative to traditional continuous endurance training (Gibala et al., 2012), inducing similar or even superior changes in numerous physiological, performance and health-related markers (e.g., Tabata et al., 1996; Helgerud et al., 2007; Wisloff et al., 2007; Gibala et al., 2012; Stöggl and Sperlich, 2014, 2015; Ni Cheilleachair et al., 2017). More specifically, HIT was shown to increase both the aerobic and anaerobic capacity (Tabata et al., 1996; Ratel et al., 2004; Gibala et al., 2006; Stöggl and Björklund, 2017) which might be of special interest for ASR as shown above. Finally, it was suggested that HIT might be more enjoyable than continuous endurance training (Tjonna et al., 2008; Bartlett et al., 2011; Lambrick et al., 2016). Therefore, HIT concepts receive special attention in sports where the time available to perform a high volume of endurance training is limited (e.g., during games sports, or based on the complex needs in ASR). For ASR, previously it was demonstrated that a short-duration HIT block (e.g., a 2 weeks shock-cycle or motor-block) was feasible and efficient to increase the VO_2max_ with 4.3–6% (Breil et al., 2010; Gross et al., 2014). It should be noted, that in both studies HIT was mainly carried out with non-skiing specific modalities like cycling or running and an obstacle course containing SL running, balancing and jumping elements.

In high performance sports, it has been suggested that improving in the quality of training is more effective in enhancing performance than just increasing the quantity of training. Several studies have suggested that testing (Mygind et al., 1991; Bilodeau et al., 1995; Helgerud et al., 2001; Stöggl et al., 2006) as well as training (Dudley and Djamil, 1985; Hakkinen and Komi, 1986; Hoff et al., 1999) should be sport specific. In specific training, the “principle of kinematic, kinetic, and neuromuscular correspondence” should be taken into consideration. This principle states that the special exercises must be in similar to those parameters of movement that characterize the structure of competition technique (Djatschkow, 1977; Menzel, 1990; Müller et al., 2000). Therefore, the specificity during training should provide coordinative affinity between training exercises and competition which results in favorable training stimuli in the relevant musculature (Müller et al., 2000).

To develop aerobic and anaerobic capacity of alpine skiers on snow, HIT would theoretically be such a specific training mode. It was recently demonstrated that by using short-radius turns during skiing, HIT efforts (>90% HR_max_ and VO_2_ values >84% VO_2max_) were possible (Stöggl et al., 2016a,b, 2017). However, the drawback to this approach is that on-snow skiing is not feasible, especially during summer, or in combination with high altitude during training phases on the glaciers. For conditioning purposes ASR specific dryland exercises may be better suited for this purpose.

Sport-specific exercises have been used in ASR with different purposes like inline skating for technical training (Zeglinksi et al., 1998; Kröll et al., 2005), specific strength training via an eccentric bike (Gross et al., 2010), or functional training by different ski ergometers (Spitzenpfeil et al., 2005; Panizzolo et al., 2013). Specific endurance training, and more specifically specific HIT, was up to now only mentioned as tool for some training sessions (three sessions out of 15; ski-specific obstacle running course) (Breil et al., 2010). Especially the ski ergometers used by Spitzenpfeil et al. (2005) and Panizzolo et al. (2013) could serve as a tool to perform controlled AS-specific HIT as they fulfill two important criteria: (1) external control of the applied load, and (2) ski specific movement instructions, based on biomechanical studies in ASR (Kröll et al., 2015a,b).

Therefore, the aims of the current study were threefold: (1) to develop different ASR specific dryland HIT protocols on a commercially available ski ergometer and to compare these with a running HIT session using physiological parameters and rating of perceived exertion (RPE); (2) determine the ski specific HIT mode, that complies best with the requirements of a HIT (e.g., intensity > 90% HR_max_), and (3) test the feasibility of a specific HIT block over 2 months with 17 training sessions as a preparation for the Pyeongchang Winter Olympics 2018 as a pilot study. We hypothesized that (a) with the ASR specific HIT protocols the intensities complying for HIT are possible and (b) with HIT using running higher physiological response will be achieved compared with the ASR specific HIT concepts.

## Materials and Methods

### Participants

Ten male junior alpine skiers were recruited from a regional Ski Gymnasium to take part in the study. Participants’ characteristics are presented in **Table 1**. The participants were healthy and competed on an international (FIS races) level. Participants and their parents were fully informed about the study details and participation requirements with written and verbal information before providing written informed consent to participate. The study received approval from the local Ethics Committee and was conducted in accordance with the Declaration of Helsinki. One participant was not able to perform all test trials based on a meniscal injury during ASR training within the period of the study.

**Table 1:** Characteristics of participants without dropouts at baseline examination (mean ± SD [Min; Max]).

Age (years)	18 ± 1 [17; 19]
Body height (cm)	190 ± 6 [171; 190]
Body weight (kg)	76 ± 9 [57.2; 88.0]
VO_2max_ (L min^-1^)	4.4 ± 0.4 [3.5; 5.0]
VO_2max_ (ml kg^-1^ min^-1^)	57.7 ± 3.7 [50.8; 62.7]
HR_max_ (bpm)	199 ± 8 [189; 218]
Peak blood lactate (mmol L^-1^)	11 ± 2 [8.4; 13.4]
FIS points slalom	40.4 ± 15.8 [15.8; 71.0]
FIS points giant slalom	40.8 ± 13.4 [16.1; 60.6]


*VO_*2*max_, maximal oxygen consumption; HR_max_, maximal heart rate; FIS, Fédération International de Ski.*

### Overall Design

Following the recruiting process every participant underwent six visits on separate days within 2 weeks during the conditioning period (June-July): (1) a VO_2max_ test running on a 400-m level outdoor track, (2) a familiarization HIT session on the ski ergometer for each of the three simulated skiing modes [that is: GS, SL, and a GS–SL mixed mode (GS/SL)], and (3) the four HIT sessions with running, GS, SL and GS/SL mixed in randomized order on four separate days with a minimum of 48 h in between. During the VO_2max_ test and each HIT session VO_2_, HR, blood lactate, RPE (BORG scale: 6–20) for the whole body (RPE_whole-body_), legs only (RPE_legs_), and arms only (RPE_arms_) were recorded. For standardization purposes, food intake was not permitted 4 h prior to testing and participants were instructed not to change their diet and amount of physical activity throughout the examination period. Furthermore, for all testing days, participants were asked to report well-hydrated and to refrain from consuming alcohol and engaging in strenuous exercise at least 24 h prior to testing.

### Ski Ergometer

A portable ski ergometer was employed for the ASR specific dryland imitation. The ergometer is based on a commercially available simulator (Pro Ski Simulator – Trgovina in storitve, Rače, Slovenia). The ergometer is equipped with six elastic bands that can be used for regulating the load during the exercise. The basic unit was reinforced to resist the high mechanical stress and assembled with a custom-manufactured slide board. The slide board was modified with an alpine ski binding to train more ski specifically. The bindings were mounted on a plate which served to lift the respective “inside heel” in order to enable ski specific knee and hip angulation (Supej, 2010). Compared with the commercial slide board “Pro Ski simulator”, the custom-manufactured ergometer was attached with clamping rolls to ensure that the board cannot derail.

**FIGURE 1:**
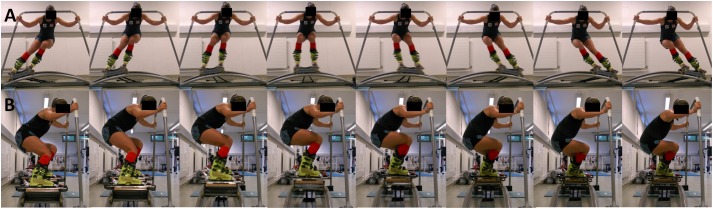
Illustrative picture series of the ski-ergometer used for the specific HIT modes giant slalom, slalom and mixed giant slalom-slalom with **(A)** frontal plane view and **(B)** sagittal plane view (Note: written informed consent was obtained from the individual for the publication of this image).

The ski-specific motion sequences on the ski ergometer were adapted to the typical on-snow SL and GS techniques based on a pilot study of Kröll et al. (2015b) and previous biomechanical studies (Supej et al., 2011; Spörri et al., 2012): The SL mode is characterized by dynamic movements with a turn cycle duration of 0.83 s and minimum extension knee angles on the outside leg of 90° at turn switch and maximum knee extension angles of about 130° between two turn switches. The GS mode is characterized by a turn cycle duration of 1.41 s and a pronounced quasi-static phase at the end of each leg extension phase. For this mode, similar knee angles to the SL mode were required, although the angles at turn switch seem to be slightly higher in real giant SL skiing (approximately 100° flexion angle). The GS/SL mixed mode alternates every interval GS and SL. To simulate representative lean angles an accompanying knee and hip angulation, participants were instructed to hold the handle bars with their hands in order to freeze the upper body in the center of the device. By doing so, arms, hands and shoulders represented a semi fixed point and just the more distal body regions oscillated against the load of the rubber bands to the left and right. The rhythm of this oscillation was provided by a digital metronome (Tempoperfect Metronome, NCH software, Greenwood Village, CO, United States) based on the above mentioned turn cycle time (SL: 72 bpm; GS: 43 bpm). The skiing specific movement execution, especially appropriate knee and hip angulation and parallel motion of the shank, was controlled by a certificated ski coach among all athletes and modes. The ski-ergometer motion is illustrated in **Figure 1**.

### VO_2max_ Test

The VO_2max_ test was performed on a level 400-m outdoor track. Each subject performed a standardized 10-min warm-up at a running speed of 8 km h^-1^. Subsequently the test protocol started with a speed of 9 km h^-1^ and a 1 km h^-1^ increment every 30 s until exhaustion. Termination criteria were either physical exhaustion, or no longer being able to keep up the running speed of the stage. Running speed was provided by a cyclist driving on the side of the runner. VO_2_ and HR were measured continuously and a lactate sample was taken during the first, third, and fifth minute upon completion of the test. VO_2max_, HR_max_, peak blood lactate, and peak running speed were determined. This test concept was not separately validated, but is a standard test that is generally applied at our institute for outdoor field tests for establishment of VO_2max_ in various sports (e.g., soccer, alpine skiing, and cross-country skiing) (e.g., Stöggl et al., 2010).

### HIT Protocols

Each of the four HIT sessions began with a 10-min warm-up with an intensity of 70% HR_max_. Participants warmed up running on the track for the running HIT protocol, and cycling on a cycle ergometer for the ski-specific HIT protocols. Each HIT session consisted of 15 bouts of 1 min each, with 30 s of low intensity active breaks, with the goal being to reach an intensity of >90% HR_max_ as quickly as possible. The 1-min interval duration was chosen in accordance with the mean run durations of the technical disciplines SL and GS (Gilgien et al., 2014), and also with reference to comparable HIT protocols presented earlier (Hawley and Gibala, 2012; Stöggl et al., 2017). For the running HIT, the intensity was paced by a cyclist (90% of peak speed during the VO_2max_ test) in combination with HR values (target HR of 90–95% HR_max_). Participants walked during the 30-s breaks. To control HR intensity, participants were instructed to reach a HR of 90–95% HR_max_ as quickly as possible. For the ASR specific HIT protocols on the ski-ergometer, the resistance provided by the number of rubber bands used was determined during the familiarization day. As criteria served the highest possible amount of rubber bands during the familiarization day the athlete could compete during repeated 1-min bouts. The 30-s breaks were performed with relaxed side-to-side bouncing on the ski-ergometer in an upright position. VO_2_ and HR were measured continuously throughout the HIT protocols. Blood lactate was sampled after the 5^th^, 10^th^, and 15^th^ interval and in the third and fifth minute post completion of the HIT protocol. Finally, RPE values for the whole-body, legs and arms were recorded upon completion of the training.

### Instruments

VO_2_ was continuously recorded by a portable breath-by-breath metabolic cart (K4b^2^, Cosmed, Italy). The athletes were fitted with a proper sized mask covering the mouth and nose (7450 Series V2^TM^ Mask, Hans Rudolph Inc., Shawnee, KS, United States). Prior to each test trial the gas analyzer’s oxygen (O_2_) and carbon dioxide (CO_2_) sensors were calibrated using a two-step calibration procedure with ambient air conditions (20.93% O_2_ and 0.03% CO_2_) and the anticipated expiratory gas percent using calibration gas containing 15% O_2_ and 5% CO_2_ (UN 1950 Aerosols, Cortex Biophysik GmbH, Leipzig, Germany) (rest volume: nitrogen). The flow volume was calibrated using a 3-L syringe. Both calibration procedures were performed directly before each test. Participants’ HR was recorded by telemetry (Suunto Ambit 3.0, Helsinki, Finland) sampling at 1-s intervals. For lactate analysis, a 20 μl capillary blood sample from the finger-tip was collected and quantified using an amperometric-enzymatic technique (Biosen S-Line Lab+, EKF-diagnostic GmbH, Magdeburg, Germany). The lactate sensor was calibrated before each test using a lactate standard sample of 12 mmol L^-1^. Results within a range of ±0.1 mmol L^-1^ were accepted.

### Eight Weeks Specific HIT Pilot Study

In a single case study during the conditioning phase (June and July), the feasibility and effects on aerobic capacity of an 8-week HIT block using the ASR specific HIT protocol on the ski-ergometers preparation for the 2018 Olympics was analyzed. One elite AS athlete (age: 25 years; height: 180 cm, weight: 85 kg, FIS points in main disciplines: Downhill 6.5 and Super-G 10.7; overall rank winner of the European Cup 2017/18) volunteered to take part. A VO_2max_ ramp protocol was performed on a cycle ergometer (Ergoline, Ergoselect 100P; Bitz, Germany) before and after (at the end of the second recovery week) the training period. The workload was set at 50 W with a 30 W increase every 30 s until exhaustion. VO_2_ was measured with a breath-by-breath spirograph as described above.

The training period consisted of two blocks of three training weeks with three HIT sessions/week (first week with only two HIT sessions) interspersed with one recovery week (only one HIT session). In total, the athlete performed 17 HIT sessions. The athlete was instructed to maintain his strength and coordination training during the intervention period. All of the HIT sessions included a 10 min warm-up on a bicycle ergometer at an intensity of 70% HR_max_, followed by the HIT protocol (15 × 1 min at > 90% of HR_max_ with 30-s recovery) alternating between the SL and GS mode between the bouts and a 10-min cool-down on the bicycle ergometer or running. Within each session HR and blood lactate values were collected (5^th^, 10^th^, and 15^th^ interval and in the third and fifth minute post completion of the HIT protocol).

### Statistical Analysis

All data exhibited a Gaussian distribution verified by the Shapiro–Wilk test and accordingly, the values are presented as means (±SD). A one-way ANOVA (four HIT protocols) was performed for each dependent variable with Bonferroni *post hoc* analysis. To compare the progression of the HR, VO_2_ and blood lactate values across the 15 intervals between the four HIT modes a 3 × 4 ANOVA with repeated measures (3 interval blocks from interval 1–5 vs. 6–10 vs. 11–15; four HIT protocols) was performed. Alpha level of significance was set to 0.05. In addition, the values obtained were evaluated by calculating the effect size (_p_η^2^) and statistical power. The Statistical Package for the Social Sciences (Version 24.0; SPSS Inc., Chicago, IL, United States) was used for statistical analysis.

## Results

### Baseline VO_2max_ Running Test

During the baseline running ramp protocol participants reached a VO_2max_ of 57.7 ± 3.7 ml min^-1^ kg^-1^ (range: 50.8–62.7) with a HR_max_ of 199 ± 8.4 bpm (189–216), and peak blood lactate values of 11.1 ± 1.0 mmol L^-1^ (8.4–13.4).

### Comparison Between Four HIT Protocols

The comparison among the four HIT protocols is presented in **Table 2**. The running HIT achieved higher physiological response in some of the measured variables in comparison to the ASR specific HIT modalities. The main differences were found with respect to cardiorespiratory parameters with higher mean VO_2_ values in the running HIT protocol compared with all three specific HIT modes (*P* = 0.008). With respect to VO_2peak_ values, running HIT was higher compared with HIT in GS (*P* < 0.001) and SL (*P* < 0.01), while no difference was found to the mixed GS/SL mode. Peak HR trended toward being lower in the GS/SL mixed HIT than the running HIT, although statistical power was low, while no differences were found for mean HR values. Peak blood lactate values was lower with HIT in GS compared with the running HIT (*P* < 0.05). RPE for the whole body was higher in the running HIT when compared with the SL and mixed GS/SL HIT mode. RPE arms was higher in GS and GS/SL mixed mode compared with the running HIT. RPE for the legs was not different between all four HIT modes.

**Table 2:** Cardiorespiratory and metabolic parameters during the high intensity training (HIT) workouts in (1) running, and on the ski ergometer with the three alpine skiing specific exercise modes, (2) giant slalom (GS), (3) slalom (SL), and (4) a mix between GS and SL (mean ± SD) (*n* = 10).

	Running	GS	SL	GS/SL mix	ANOVA			
HR_mean_ (bpm)	182 ± 10	175 ± 9	175 ± 7	173 ± 7	*F*_3,6_ = 4.4	*P* = 0.058	_p_η^2^ = 0.69	
HR_peak_ (bpm)	195 ± 11	191 ± 12	191 ± 9	189 ± 9^∗^	*F*_3,6_ = 5.2	*P* = 0.041	_p_η^2^ = 0.72	pow = 0.67
Rel. HR_mean_ (% HR_max_)	91 ± 2	88 ± 2	88 ± 3	87 ± 4	*F*_3,6_ = 4.5	*P* = 0.055	_p_η^2^ = 0.69	
Rel. HR_peak_ (% HR_max_)	98 ± 2	96 ± 3	96 ± 3	95 ± 3^∗^	*F*_3,6_ = 5.3	*P* = 0.040	_p_η^2^ = 0.73	pow = 0.67
VO_2mean_ (ml kg^-1^ min^-1^)	45 ± 3	40 ± 4^∗∗^	41 ± 3^∗∗^	41 ± 4^∗^	*F*_3,6_ = 10.5	*P* = 0.008	_p_η^2^ = 0.84	pow = 0.93
VO_2peak_ (ml kg^-1^ min^-1^)	56 ± 3	51 ± 4^∗∗∗^	52 ± 4^∗∗^	53 ± 5	*F*_3,6_ = 31	*P* < 0.001	_p_η^2^ = 0.94	pow = 1.00
Rel. VO_2mean_ (%VO_2max_)	79 ± 5	68 ± 4^∗∗^	71 ± 5^∗∗^	70 ± 5^∗^	*F*_3,6_ = 10.9	*P* = 0.008	_p_η^2^ = 0.85	pow = 0.94
Rel. VO_2peak_ (%VO_2max_)	97 ± 3	88 ± 4^∗∗∗^	91 ± 4^∗∗^	91 ± 5	*F*_3,6_ = 32	*P* < 0.001	_p_η^2^ = 0.94	pow = 1.00
Peak blood lactate (mmol L^-1^)	9.6 ± 2.8	6.3 ± 2.2^∗^	6.5 ± 1.6	5.9 ± 1.4	*F*_3,6_ = 4.6	*P* = 0.053	_p_η^2^ = 0.70	
RPE_wholebody_	18.2 ± 0.8	16.6 ± 2.0	16.3 ± 2.1^∗^	16.1 ± 1.5^∗∗^	*F*_3,6_ = 26	*P* = 0.001	_p_η^2^ = 0.93	pow = 1.00
RPE_arms_	13.3 ± 2.2	16.4 ± 2.2^∗∗^	15.1 ± 2.5	16.2 ± 2.2^∗^	*F*_3,6_ = 6.6	*P* = 0.025	_p_η^2^ = 0.77	pow = 0.77
RPE_legs_	16.2 ± 1.6	16.6 ± 1.0	16.4 ± 1.9	15.9 ± 0.9	*F*_3,6_ = 1.1	*P* = 0.406	_p_η^2^ = 0.36	


*HR, heart rate; VO_*2*_, oxygen uptake; VO_*2mean*_, mean oxygen uptake across the HIT protocol; VO_*2peak*_, peak oxygen uptake within the respective HIT protocol; RPE, rate of perceived exertion; ^∗^*P* < 0.05; ^∗∗^*P* < 0.01; ^∗∗∗^*P* < 0.001, significantly different to HIT Running; _*p*_η^*2*^, partial eta square effect size; pow, statistical power.*

**FIGURE 2:**
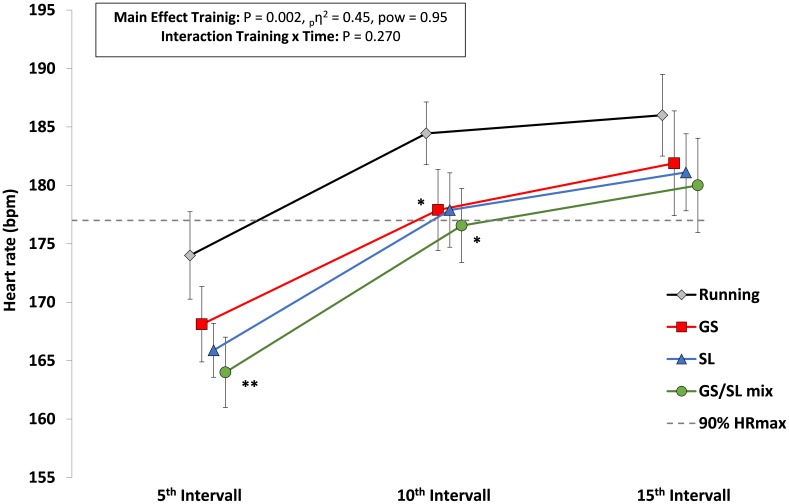
Peak heart rate values within interval 1–5 (5^th^ interval), 6–10 (10^th^ interval), and 11–15 (15^th^ interval) for the four HIT protocols with running, giant slalom mode (GS), slalom mode (SL), and the mixed GS/SL mode. ^∗^*P* < 0.05, ^∗∗^*P* < 0.01, significant different to running; _p_η^2^, partial eta square effect size; pow, statistical power; and mean ± SD.

**FIGURE 3:**
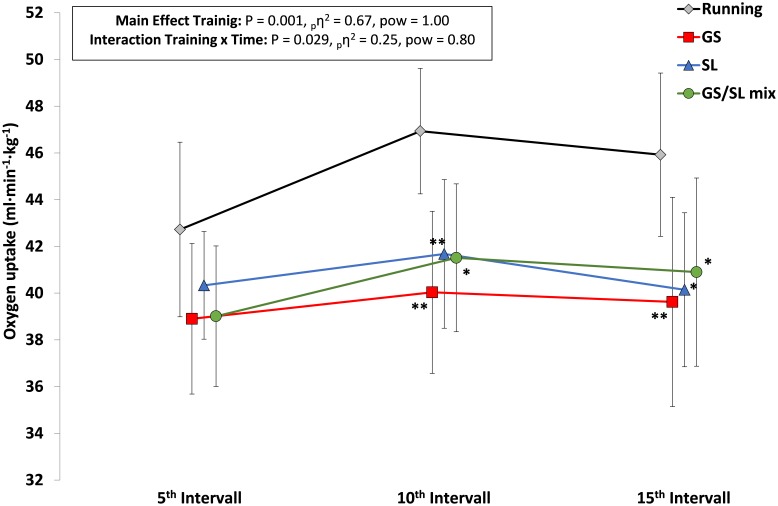
Peak VO_2_ values within interval 1–5 (5^th^ interval), 6–10 (10^th^ interval), and 11–15 (15^th^ interval) for the four HIT protocols with running, giant slalom mode (GS), slalom mode (SL), and the mixed GS/SL mode. ^∗^*P* < 0.05, ^∗∗^*P* < 0.01, significant different to running; _p_η^2^, partial eta square effect size; pow, statistical power; and mean ± SD.

**FIGURE 4:**
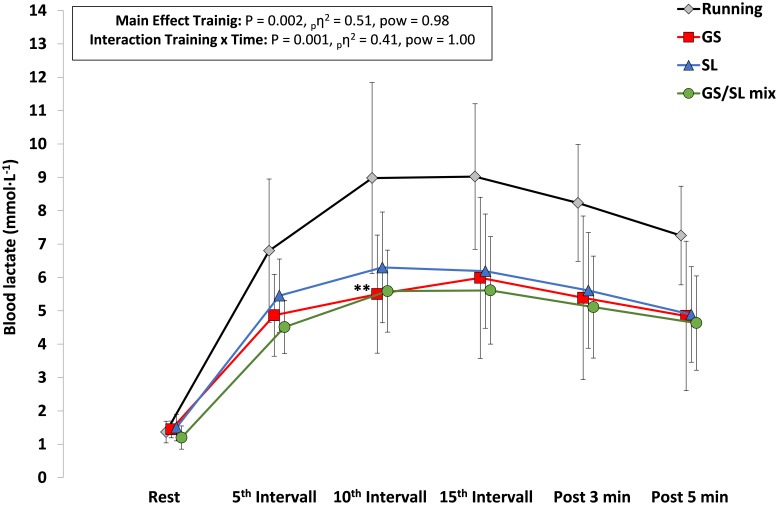
Blood lactate values after the 5^th^, 10^th^, and 15^th^ interval and in the third and fifth minute post exercise for the four HIT protocols with running, giant slalom mode (GS), slalom mode (SL), and the mixed GS/SL mode. ^∗∗^*P* < 0.01; significant different to running; _p_η^2^, partial eta square effect size; pow, statistical power; and mean ± SD.

An illustration of the evolution of HR, VO_2_ and blood lactate values across the 15 1-min intervals within each HIT session is presented in **Figures 2–4**. Interaction effects for time x HIT mode were found for VO_2_ (*P* = 0.029) and blood lactate (*P* = 0.001) and with a more pronounced increase across the training session in the running HIT compared with the ASR specific HIT protocols.

### Pilot Training Study

**FIGURE 5:**
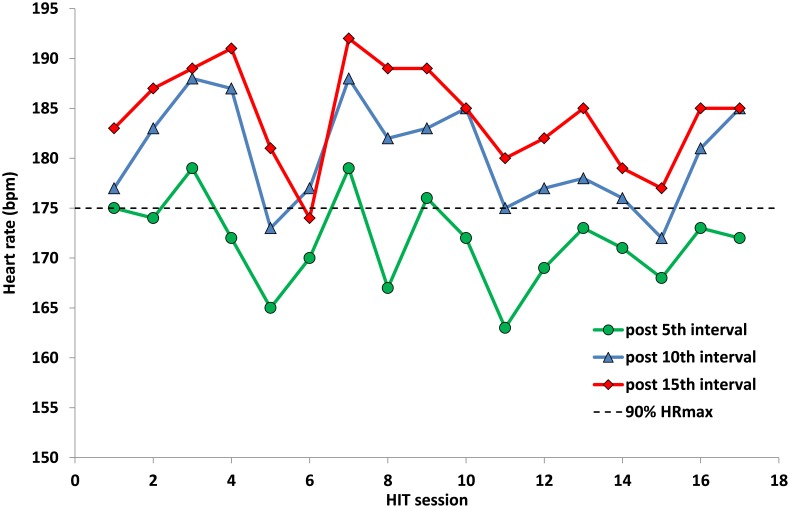
Peak heart rate values after the 5^th^, 10^th^, and 15^th^ interval across the 17 HIT sessions during the 8-week training period.

**Figure 5** illustrates the development of HR values across the 17 HIT sessions within the 8 weeks training period. All the HIT sessions could be easily performed by the athlete and he was able to reach in all the training sessions a HR > 90% HR_max_, mostly after the 10^th^ interval. Peak blood lactate was 8.1 ± 1.3 mmol L^-1^ (range: 6.5–11.6) across the 17 training sessions. Following the HIT intervention the VO_2peak_ was increased by 11% (Pre: 49.0, Post 54.5 ml min^-1^ kg^-1^) and the peak power output during cycling remained constant at 530 W.

## Discussion

The main findings of the current study are: (1) all four HIT protocols induced sufficient physiological loading to be quantified as HIT (e.g., >90% HR_max_, >88% VO_2max_, blood lactate > 5.9 mmol L^-1^); (2) physiological response with respect to VO_2_ values was higher in the running HIT compared with the three ASR specific HIT modes; (3) the three ASR specific HIT modes did not differ with respect to the analyzed physiological and RPE values; (4) RPE for the whole body was higher during the running HIT when compared with the specific HIT SL and HIT GS/SL; (5) RPE for the arms was more pronounced in the three ASR specific HIT modes compared with running HIT while no difference was found for RPE legs; and (6) a pilot study in one participant using an ASR specific HIT program over 8 weeks was shown to be feasible and led to an 11% improvement in VO_2max_ with unchanged peak power output during a cycling ramp protocol.

In various measured parameters the running HIT led to higher physiological response compared with the ASR specific HIT modes. This was especially true for the VO_2_ values and with respect to selected ASR specific modes for HR (e.g., GS/SL mixed), peak blood lactate (GS), and whole-body RPE (SL and GS/SL mixed). However, all four HIT protocols still reached clearly the intensity criteria for being classified as HIT (e.g., Stöggl and Sperlich, 2015) (all four modes: HR_peak_ > 90% HR_max_, VO_2peak_ > 0.88% VO_2max_; blood lactate values >5.9 mmol L^-1^). In addition, the intensity during the specific HIT protocols on the ski-ergometer was comparable with reported values achieved during actual ASR on-snow training and competition. As stated earlier, %VO_2max_ values of 64–100% and %HR_max_ values of 89–102% are documented during ASR (Karlsson et al., 1978; Veicsteinas et al., 1984; Saibene et al., 1985; Tesch, 1995; Vogt et al., 2005; Grenier et al., 2013; Polat, 2016). Specifically, within the three ASR specific HIT modes a pronounced physiological response was attained (95–96% HR_max_ and 88–91% VO_2max_) and participants were able to maintain the intensity across the 15 intervals of each HIT protocol (total duration of 22 min of HIT including the 30 s breaks). Most likely, these high values during the specific HIT modes can just be attained if high strength and coordinative capacities of the athletes are already developed. As demonstrated during ASR, these are the prerequisites of elite alpine athletes to enable them to ski in a very active and exhausting skiing style (Karlsson et al., 1978; Berg and Eiken, 1999; Neumayr et al., 2003).

Except for one significant difference in peak HR between GS/SL mixed HIT vs. running HIT no clear differences in HR could be detected between the four HIT modes. However, this significant difference needs to be assessed critically based on the low statistical power. Based on the high effect size it can be assumed that with a higher sample size the running HIT might have led to higher physiological load also with respect to HR compared with the ASR specific HIT modes. VO_2_ values were higher in the running HIT protocol – particularly when compared with the pure SL and GS modes. This could possibly be based upon the differences in the mechanics of the muscular work during the running vs. ski specific ski ergometer exercise. Alpine skiing is characterized by a mix of static and dynamic muscle activity of the lower extremities (Müller and Schwameder, 2003; Kröll et al., 2010) with both moderate to high concentric and eccentric loading (Berg et al., 1995; Tesch, 1995; Berg and Eiken, 1999; Kröll et al., 2015a). In contrast to that, mainly dynamic and cyclic muscle loading occurs during other exercises like cycling and running. It might be speculated, that this type of muscle loading during the ASR imitation exercise resulted in the attenuated increase in cardiorespiratory output based on muscular limitations when compared to running (Stöggl et al., 2016a,b, 2017). In this context, in a study in cross-country skiing it was suggested that O_2_ extraction can be attenuated based on mechanical hindrance, like magnitude and pattern of force application, muscle activation patterns and time coordination between loading and unloading within a cyclic motion (Stöggl et al., 2013). This attenuation in physiological output could also have been observed in the less pronounced rise in VO_2_ and blood lactate values during the three ASR specific HIT modes compared with the running HIT (**Figures 1, 2**). The interaction effects demonstrated that this rise was lower within the first 10 intervals. Therefore, the initial intervals within the ASR specific HIT modes need to be more intense, but merits further analysis.

With respect to the subjective loading of the four HIT protocols, it was found that the legs were equally loaded in all HIT protocols. Whole body RPE was highest during the running HIT, and especially greater compared with the SL and SL/GS HIT modes.

In a study of fit and unfit recreational skiers it was demonstrated that the subjective loading of the legs during alpine skiing was even greater when compared with classical endurance exercises like cross-country skiing and cycling (Stöggl et al., 2016a,b, 2017) – however, no comparison to running was performed. One would presume that a strength-oriented task like the ski ergometer would result in perceived higher demands for the legs compared to the more endurance-oriented task running. ASR athletes usually train their aerobic capacity more on cycle ergometers than with running (Breil et al., 2010), however, they perform a lot of strength and strength endurance training with knee-hip-extension-flexion exercises like squats or jumps in different forms (Patterson et al., 2009). Therefore, the relative inexperience of the ASR athletes in running serves as an explanation for relative high leg RPE values not only for the specific HIT modes but also during running.

The use of the arms during the ski ergometer exercise led to increased arm RPE when compared to the running HIT. The athletes were instructed to hold the handle bars with their hands in order to freeze the upper body in the center of the device. By doing so a simulation of representative lean angles and accompanying knee and hip angulation was possible (Supej, 2010). Therefore, the ski specific exercise leads to a clear loading, and perceived exhaustion of the arms. To create the movement on the ski ergometer (i.e., produce force against the rubber bands), the participants used the arms and the upper body to generate force which was transmitted down the kinetic chain to the skiing platform. Therefore, it seems plausible that this leads to distinct and specific activation of the trunk for the stabilization and control of the motion, especially during the static hold at the end points of the GS mode. With respect to injury prevention the aspect of adequate stimuli for core stability by using the ski-ergometer could be of interest from two perspectives: The prevention of low back pain overuse injuries but also for the prevention of acute knee injuries which both are described as serious problem in ASR (Florenes et al., 2009; Spörri et al., 2015). For both areas insufficient core strength, respectively, stability seems to be a main risk factor. Raschner et al. (2012) suggested that core strength is a predominant critical factor for ACL injuries in young ski racers.

Consequently, the ASR specific ski-ergometer exercise described in the current study might constitute a good combination to specifically train HIT, but also to specifically load and train important structures and muscles for ASR with respect to performance (i.e., high leg RPE values) but also specific injury prevention (core stability). Future research is needed to prove this concept on effectivity in enhancing ASR performance.

All specific HIT modes led to comparable physiological and metabolic loading, whether cycle frequency was high (e.g., SL) or low (e.g., GS) or if these two modes were alternately applied during each consecutive interval (GS/SL mix). Though not statistically significant, VO_2_ was higher and whole-body RPE lower in the GS/SL mixed mode than the GS or SL HIT. Based on this outcome, as well as the feedback from athletes preferring less monotonously sessions, the GS/SL mixed HIT protocol was applied for a single case pilot training study in one elite ASR skier to check if this training concept is feasible for ASR elite sports. The results demonstrate that the athlete was able to perform all 17 sessions with sufficient physiological response (>90% HR_max_ in every session and mean peak blood lactate values of 8.1 mmol L^-1^), with no marked signs of overloading symptoms. The training led to an 11% increase in the VO_2max_ and unchanged peak power output on the cycle ergometer. Based on these findings, a future study is warranted demonstrating if the implementation of a specific HIT over a longer period of time is effective in increasing aerobic and anaerobic capacity, peak power output and specific strength of the lower body and the trunk when compared with a standard HIT training concept in running or cycling.

## Conclusion

The HIT protocol with running resulted in a more pronounced response in VO_2_ compared with the three ASR specific HIT modes. However, with all four HIT protocols participants were able to reach exercise intensities sufficiently high to be classified as HIT (>90% HR_max_ and >88% VO_2max_). The increased loading of the arms during the ASR specific HIT workouts might be of special interest for training both the aerobic and anaerobic capacity but also upper body and trunk strength, all important aspects or performance and injury prevention in ASR. Consequently, the combination of the ASR specific ergometer with a 15 × 1-min HIT protocol might provide sufficient stimulus for the cardiorespiratory and metabolic systems to enhance aerobic and anaerobic capacity. The effectiveness of such a HIT protocol over longer duration was shown to be feasible in one elite alpine skier. Based on his successful skiing season following this intervention (e.g., winner of the European Cup total ranking in downhill skiing), no negative side-effects on true skiing capabilities can be stated so far. However, the effects of this training protocol on a larger group of elite alpine skiers on aspects of endurance, power and core needs to be proven in a future study. Furthermore, a detailed description of the functional (biomechanical) similarity between the instructed movement on the ski-ergometer and the on-snow situation would complete the picture on how the ergometer could be used best possible in the off-season training of ASR athletes.

## Author Contributions

TS, JK, RH, MC, and EM conceived and designed the experiments, and read and approved the final manuscript. JK, RH, and MC performed the experiments. TS, RH, and MC analyzed the data. TS, JK, and RH prepared the manuscript.

## Conflict of Interest Statement

The authors declare that the research was conducted in the absence of any commercial or financial relationships that could be construed as a potential conflict of interest.
